# Growth differentiation factor-15 and circulating biomarkers as predictors of periodontal treatment effects in patients with periodontitis: a randomized-controlled clinical trial

**DOI:** 10.1186/s12903-023-03237-y

**Published:** 2023-08-21

**Authors:** Gaetano Isola, Gianluca Martino Tartaglia, Simona Santonocito, Akhilanand Chaurasia, Anand Marya, Antonino Lo Giudice

**Affiliations:** 1 Department of General Surgery and Surgical-Medical Specialties, School of Dentistry, University of Catania, Via S. Sofia 78, Catania, 95123 Italy; 2 Department of Biomedical, Surgical and Dental Sciences, School of Dentistry, University of Milan, Milan, 20100 Italy; 3 Ospedale Maggiore Policlinico, Fondazione IRCCS Cà Granda, Milan, 20100 Italy; 4 Department of Oral Medicine, Faculty of Dental Sciences, King George’s Medical University, Lucknow, Uttar Pradesh India; 5 Department of Orthodontics, University of Puthisastra Phnom Penh Combodia, Phnom Penh, 55180 Cambodia; 6 Center for Transdisciplinary Research, Saveetha Dental College, Saveetha Institute of Medical and Technical Science, Saveetha University, Chennai, 600077 India

**Keywords:** Periodontitis, Treatment outcome, Growth differentiation factor 15, Related circulating systemic risk, Glutathione peroxidase 1, c-reactive protein, And surfactant protein D, Serum, Periodontal debridement, Full mouth disinfection, Clinical trial

## Abstract

**Background:**

During the last decades, in patients with periodontitis, periodontal treatment has been shown to reduce the potential release of local and systemic biomarkers linked to an early risk of systemic inflammatory disorders. This study evaluated the efficacy of non-surgical-periodontal treatment (NSPT) on growth differentiation factor 15 (GDF-15) and related circulating biomarkers such as glutathione peroxidase 1 (GPx-1), c-reactive protein (hs-CRP), and surfactant protein D (SP-D) in periodontal patients and explored whether subjects who had high GDF-15 levels at baseline showed increased clinical benefits following NSPT at 6-months follow-up.

**Methods:**

For this two-arm, parallel randomized clinical trial, patients with periodontitis were randomly allocated to receive quadrant scaling and root-planing (Q-SRP, n = 23, median age 51 years old) or full-mouth disinfection (FMD, n = 23, median age 50 years old) treatment. Clinical and periodontal parameters were recorded in all enrolled patients. The primary outcome was to analyse serum concentrations changes of GDF-15 and of GPx-1, hs-CRP, and SP-D at baseline and at 30, 90, and 180-days follow-up after NSPT through enzyme-linked immunosorbent assay (ELISA) and nephelometric assay techniques.

**Results:**

In comparison with FMD, patients of the Q-SRP group showed a significant improvement in clinical periodontal parameters (p < 0.05) and a reduction in the mean levels of GDF-15 (p = 0.005), hs-CRP (p < 0.001), and SP-D (p = 0.042) and an increase of GPx-1 (p = 0.025) concentrations after 6 months of treatment. At 6 months of treatment, there was a significant association between several periodontal parameters and the mean concentrations of GDF-15, GPx-1, hs-CRP, and SP-D (p < 0.05 for all parameters). Finally, the ANOVA analysis revealed that, at 6 months after treatment, the Q-SRP treatment significantly impacted the reduction of GDF-15 (p = 0.015), SP-D (p = 0.026) and the upregulation of GPx-1 (p = 0.045).

**Conclusion:**

The results evidenced that, after 6 months of treatment, both NSPT protocols improved the periodontal parameters and analyzed biomarkers, but Q-SRP was more efficacious than the FMD approach. Moreover, patients who presented high baseline GDF-15 and SP-D levels benefited more from NSPT at 6-month follow-up.

**Trial registration:**

NCT05720481.

**Supplementary Information:**

The online version contains supplementary material available at 10.1186/s12903-023-03237-y.

## Introduction

Periodontitis is a chronic, multifactorial, inflammatory disease caused by infectious biofilm that, if not prevented and treated appropriately, might destroy the tooth-supporting tissues and cause tooth loss [1]. During the last few decades, several large-cohort studies have reported that periodontitis may afflict more than half of the world’s population and negatively impact a number of systemic disorders, including cardiovascular disease (CVD) [2], diabetes [3], metabolic syndrome [4] and that lowering the overall quality of life [5]. In this regard, some evidence suggests that the development and progression of periodontitis is associated with the dysregulation of certain pro-inflammatory mediators, including interleukins (Ils), prostaglandins, c-reactive proteins (hs-CRP) and other inflammatory mediators that are released into the bloodstream during the active stages of the disease [6]. This might indicate an elevated risk of chronic systemic inflammation, endothelial dysfunction, and CVD through specific oxidative stress routes [7, 8]. In this regard, a significant association between periodontitis, heart failure and CVD has been assessed in several large population studies in which periodontitis patients were more commonly related to the early risk of myocardial infarction and endothelial dysfunction risk [9, 10].

Recently some cohort clinical trials have shown that the growth differentiation factor 15 (GDF-15), a biomarker released in cardiac and blood wall tissues in response to heart volume expansion or abnormal pressure load, is associated with early diagnosis and bad outcomes in patients with CVD and heart failure [11]. Evidence suggests that elevated serum GDF-15, together with 1-antitrypsin and glutathione peroxidase 1 (GPx-1) levels, are associated with a high risk of acute coronary syndrome, a phenomenon that occurs just during early heart failure in asymptomatic patients and for the risk stratification of patients with the acute coronary syndrome, endothelial dysfunction [12, 13]. Furthermore, in some large-population studies, GDF-15 and GPx-1 have been shown to impact various outcomes of CVD and atrial fibrillation [14, 15].

In periodontitis patients, previous reports have shown that upregulated GDF-15 levels sustain the inflammatory response of periodontal ligament fibroblasts against *P. gingivalis* [16] and orchestrate the extent of periodontal tissue destruction already in the early stage of the disease [17]. In this regard, a number of NSPT protocols have been reported that periodontal treatment might reduce certain CVD biomarkers [18]. More specifically, in patients with periodontitis, the NSPT performed through full-mouth disinfection (FMD) protocol, conducted with the adjuvant use of chlorhexidine, was shown to be more successful than traditional quadrant scaling and root planing (Q-SRP) in decreasing various inflammatory biomarkers, such as hs-CRP [19], particularly in the short-term run [20–22].

Finding mediators that might improve the predictive accuracy of systemic inflammatory risk and assessing the impact of periodontal therapy on early indicators of CVD and endothelial dysfunction are of increasing interest. In this regard, the purpose of the present randomized clinical trial (RCT) was to evaluate the effect of NSPT performed either through Q-SRP or FMD on GDF-15, GPx-1, hs-CRP, and SP-D concentrations in patients with periodontitis at 6-month follow-up after treatment. In addition, it was analyzed the impact of both NSPT protocols on serum GDF-15, GPx-1, hs-CRP, and SP-D, and if patients who harboured high baseline GDF-15 and related biomarkers levels benefited more from the efficacy of NSPT. The null hypothesis to invalidate was that, at the 6-month follow-up, there were no significant changes in serum GDF-15, GPx-1, hs-CRP, and SP-D among enrolled patients treated with different NSPT and that baseline biomarkers did not impact the efficacy of NSPT.

## Materials and methods

### Study design and sample

The present RCT was performed according to the 2016 Helsinki Declaration on medical research. The ethical approval was obtained from the International Review Board of the University of Catania, Catania, Italy (22–149 PO). All participants signed the research informed consent; the study was retrospectively registered on clinicaltrials.gov (NCT05720481–09/02/23). The study was conducted in accordance with the CONSORT guidelines [23].

For the present RCT, consecutive patients with a diagnosis of periodontitis [24] were recruited at the Unit of Periodontology of the Dental School of the University of Catania, Catania, Italy, from September 2019 to September 2022 in order to receive NSPT performed either with Q-SRP or FMD protocol. At baseline, 208 patients with periodontitis were first screened; in order to obtain an equal age and gender proportion, were enrolled male and female patients aged between 30 and 65 years ensuring that at least 50% of them were female.

The inclusion criteria were (1) good general health, (2) a minimum of six teeth per quadrant, (3) at least two teeth in each quadrant with a probing depth (PD) ≥ 5 mm and a clinical attachment loss (CAL) ≥ 4 mm, (4) at least 40% of periodontal sites with bleeding on probing (BOP), and (5) at least 2 sites with radiographically verifiable alveolar bone loss (ABL) [25]. The exclusion criteria were as follows: (1) any periodontal therapy in the previous 12 months prior to the study; (2) tooth with furcation involvement; (3) use of antibiotics in the previous 6 months prior to the study; (4) status of pregnancy or lactation; (5) presence of any systemic disease that could affect the study results; (6) use of mouthwash containing antimicrobials in the previous 3 months prior to the study; (7) use of anti-inflammatory, immunosuppressive, or contraceptive drugs; (8) alcohol consumption.

After the identification of each eligible participant, medical history, demographic aspects such as age, gender, body mass index (BMI), comorbidities (if present), medicines, and levels of education recording of PD, BOP, gingival recession, and plaque index score was carried out. BMI (kg/m^2^) was obtained by dividing the patient’s weight by the cube of their height. In addition, the patient’s socioeconomic status (SES), as an economic and sociological combined total measure of a work experience and of an individual’s or family’s economic access to resources and social position in relation to others, was annotated considering a high moderate, or low SES [26]. On the basis of patient’s smoking history, each participant was categorized as a current smoker, ex-smoker (patients who stopped smoking ≥ 5 years), and non-smoker.

Subsequently, all recruited patients received a comprehensive dental and oral examination performed by 2 masked examiners. The periodontal examination was achieved using a conventional periodontal probe at six sites per tooth (UNC-15, Hu-Friedy, Italy), recording PD, BOP [27], and plaque index score (PI) [28]. CAL was calculated by measuring PD and the amount of gingival recession from the cementoenamel junction (CEJ) to the gingival margin. ABL was evaluated on Rinn X-rays at the interproximal tooth surface by measuring the distance from the CEJ to the alveolar crest (AC).

### Sample size and reliability analysis

The power sample analysis, calculated using statistical software (G* POWER, Universität Düsseldorf, Germany), was obtained by setting up serum GDF-15 as a primary outcome variable [11] and considering two groups of patients, an effect size of 0.30, a 2-sided level of 0.05, a standard deviation of 1.5, and a power level of 80%. Therefore, it was fixed a priori that at least 22 patients per group were needed to achieve an adequate power sample. However, to avoid potential dropouts during the 6-month follow-up, 23 patients were enrolled, so the primary variable (CAL) achieved a power value of 0.80.

An inter-examiner reliability test among examiners (S.S., A.L.G.) was performed using Cohen’s kappa coefficient and showed an agreement of 85.8% (k = 0.63) for the primary outcome chosen, CAL, indicating a high degree of reliability. The kappa coefficient was also calculated for the measurements taken at each follow-up session, and an acceptable degree of reliability was established for every examination (intra-class correlation coefficient, ICC = 0.770).

### Randomization

Through a permuted block design, the randomization was performed by a single clinician, not involved in the subsequent trial stages, which generated a random assignment of a treatment using a sequence 1:1 ratio by a computer random-number generator.

Each patient was allocated to receive Q-SRP or FMD. The allocation was concealed to the clinician who performed the NSPT protocol using serially numbered, sealed envelopes, and the sequence details were concealed from all other clinicians. Before each treatment, a clinician who was not engaged in data processing assigned the patient’s treatment to a sealed envelope containing the treatment’s name and initials.

Just prior to each treatment session, a clinician not involved in the subsequent study stages opened the envelope containing the patient’s assigned therapy which he handed to the clinician for treatment. To eliminate bias in the experimental data, all the procedures were performed by the same blinded clinician with ten years of expertise in periodontics.

### Study outcomes

The primary outcome was the analysis of serum GDF-15 expression changes between groups after 6 months following NSPT protocols. Furthermore, the impact of NSPT on GPx-1, hs-CRP, and SP-D concentration changes was analysed after 6 months of treatment. The secondary objective was to examine the influence and interaction between NSPT protocol (Q-SRP used as a reference) and the duration of NSPT (6 months) on GDF-15, GPx-1, hs-CRP, and SP-D changes, as well as whether high baseline levels of GDF-15, GPx-1, hs-CRP, and SP-D influenced the efficacy of periodontal treatment after 6 months of follow-up.

### Treatment

Shortly after the baseline assessments, each enrolled patient received oral hygiene instructions. Patients allocated to the FMD group received a full mouth SRP in one side of the mouth for each session, within 24 h in two separate sessions, on two consecutive days with the adjunctive use of local antiseptic in accordance with the protocol of *Quirynen et al.* [29]. SRP was performed using both hand and ultrasonic tips instrumentation No. 5/6/7 (Satelec Ultrasonics, Acteon, VA, Italy), used with constant water irrigation with a 20.000 Hz frequency. Two right quadrants were instrumented in the morning, while the other two were instrumented in the afternoon, along with brushing the dorsum of the tongue with 1% chlorhexidine gel for 1 min, rinsing twice with 0.2% chlorhexidine mouthwash solution for 1 min, applying 0.2% chlorhexidine spray (GlaxoSmithKline, Milan, Italy) twice per day to the tonsils, and by performing subgingival irrigation of all periodontal pockets with 1% chlorhexidine gel (three times within 5–10 min), which was repeated one week later. Patients were instructed to rinse twice a day for 1 min with 0.2% chlorhexidine solution and to spray the tonsils twice a day with 0.2% chlorhexidine spray over a period of 2 months.

Patients allocated to the Q-SRP group received quadrant SRP in four different sessions with an interval of 1 week. In each patient, the first session started in the upper right maxillary quadrant. Treatments were recorded in minutes and performed under local anaesthesia only if necessary. At the conclusion of each type of treatment, all patients undergo to periodontal supportive therapy in which each patient was motivated to reinforce domiciliary oral hygiene measures.

### Sampling

At baseline and at 3- and 6 months after NSPT, blood samples from each patient were taken between 8:00 and 10:00 a.m., before any periodontal examination. Following sampling, serum samples were centrifuged at 4 °C (1000x g for 2 min) and stored. The serum GDF-15, GPx-1, and SP-D concentration levels were obtained using a specific kit according to the manufacturer’s instructions and were evaluated using human-specific enzyme-linked immunosorbent assay (ELISA) kits. The hs-CRP levels were obtained by a nephelometric assay kit.

### Statistical analysis

Numerical data were expressed by mean ± standard deviation (SD), while categorical variables were reported as numbers and percentages. Because most of the analyzed variables were not normally distributed, as verified by the Kolmogorov-Smirnov test, a non-parametric approach was applied. The Mann-Whitney test was used for the numerical data comparison between groups, while the Chi-Square test was applied for the comparisons between categorical variables. The single patient was set as a test unit. For intragroup comparisons, the Friedman test was applied to compare numerical variables over four-time intervals (baseline, 30, 90, and 180 days), whereas the Wilcoxon test was used for two-by-two comparisons across dependent groups. Bonferroni’s correction was applied for multiple comparisons, and the alpha level of 0.050 was set and was divided by the number of potential comparisons (baseline, 3 months, 6 months) to get an adjusted significance level of 0.017 (0.050/3). The Spearman’s correlation test was used to examine a potential substantial dependency between GDF-15, GPx-1, hs-CRP, and SP-D and all analyzed variables at a 6-month follow-up.

To analyze the effect of the treatment protocols on GDF-15, GPx-1, hs-CRP, and SP-D (continuous variables), a two-way ANOVA was used to estimate whether the mean of the quantitative variable (GDF-15, GPx-1, hs-CRP, and SP-D) changes based on the levels of two categorical variables, treatment and timing of treatment. Specifically, it was evaluated how the two independent variables (treatment protocols and timing), alone and in combination, influenced serum GDF-15 concentration changes. The same models were applied for the secondary outcomes GPx-1, hs-CRP, and SP-D changes. Statistical analyses were performed using IBM SPSS version 22 Statistical software for Windows (Armonk, NY, IBM corp). A significant P-value was set as < 0.05.

## Results

### Patient characteristics

**Fig. 1:**
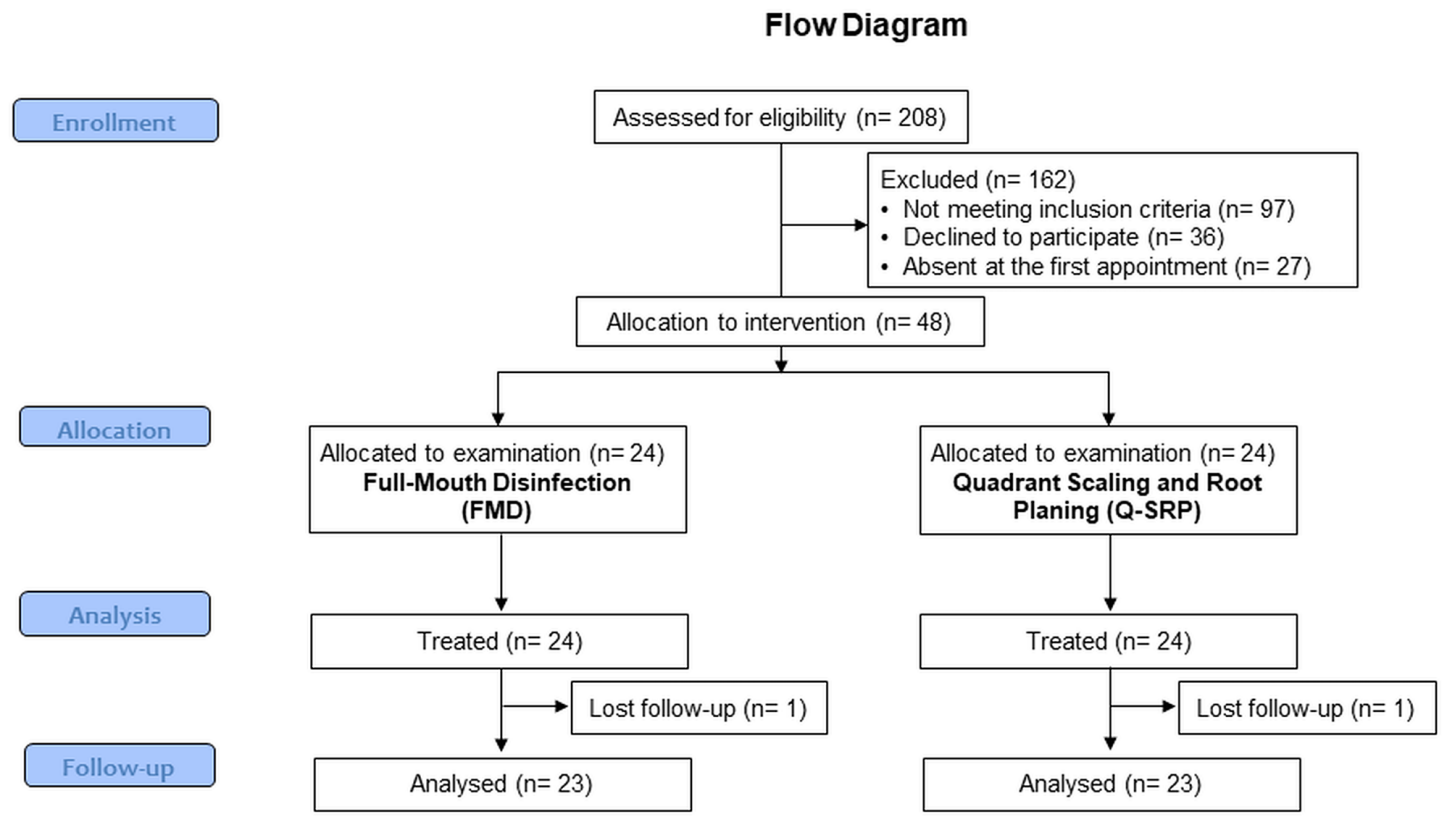
Workflow of the study

Following the first patient screening, a total of 162 patients were finally excluded because they did not fully meet the study criteria (n = 97), refused to participate in the study (n = 36) or were absent at the periodontal examination at baseline (n = 27). Two patients in each group were lost during the follow-up sessions, and a total number of 46 patients with stage III periodontitis were finally analyzed (Fig. 1).

In both groups, gender (p = 0.331), age (p = 0.596), HbA1c (p = 0.541), lipids (total cholesterol, LDL and HDL cholesterol) and the number of smokers (p = 0.417) were well matched (Table 1). There was no difference in the NSPT duration of treatment between the Q-SRP (41.3 ± 5.7 min) and FMD (39.8 ± 4.6 min) groups (p = 0.247).

**Table 1:** Baseline characteristics of the research sample. Values are reported as frequency, median and IQR, and interquartile range (1st;3rd). IQR, Interquartile Range; BMI, Body Mass Index

Characteristics	FMD (n = 23)	Q-SRP (n = 23)	P-value
Male/female, n°	10/13	11/12	0.331
Age, median (IQR)	50 (48.6–52.4)	51 (47.6–54.9)	0.596
*Race*			0.912
Caucasians, n. (%)	21 (91.3)	21 (91.3)	
Black, n. (%)	2 (8.7)	2 (8.7)	
*Education level*			0.326
Primary School, n. (%)	11 (47.8)	12 (52.2)	
High School, n. (%)	7 (30.4)	7 (30.4)	
University, n. (%)	5 (21.8)	4 (17.4)	
HbA1c %, median, (IQR)	5.1 (4.8–5.2)	5 (4.7–5.2)	0.541
Total cholesterol (mg/dl)	175 (122; 189)	172 (142–191)	0.332
HDL cholesterol (mg/dl)	42 (35–49)	43 (32–51)	0.285
LDL cholesterol (mg/dl)	78 (71–86)	79 (68–93)	0.105
BMI (kg/m^2^), median, (IQR)	20.7 (18.2–22.1)	20.2 (17.7–23.1)	0.425
*Smoking*			0.417
Current smokers, n. (%)	1 (4.4)	1 (4.4)	
Former smokers, n. (%)	-	1 (4.4)	
Non-smokers, n. (%)	22 (95.6)	21 (91.2)	
Teeth at baseline median, (IQR)	21 (17.8–24.4)	22 (18.7–23.9)	0.254

Table 2 shows the periodontal features of the study groups. Compared with FMD, treatment with Q-SRP determined, at 6-months after treatment, a significant decrease in mean PD (p = 0.013), mean CAL (p = 0.015), in the reduction of % PD ≥ 4 mm (p < 0.001) and in the reduction of BOP (p = 0.016) (Table 2).

**Table 2:** Characteristics of the sample’s periodontium at baseline and at each follow-up visit. Values are reported as mean ± standard deviation (SD). PD, Probing Depth; CAL, Clinical Attachment Loss; BOP, Bleeding on Probing; PI, Plaque Index

Variable	FMD (n = 23)	Q-SRP (n = 23)	P-value
**PD, mm**			
Baseline	4.65 (3.96; 5.19)	4.72 (4.21; 5.26)	0.524
1-month	3.97 (3.78; 4.12) ^b^	3.47 (3.32; 3.75) ^b^	0.044
3-months	3.76 (3.61; 3.88) ^c^	3.04 (2.96; 3.39) ^c^	0.025
6-months	3.39 (3.28; 3.58) ^d^	2.79 (2.68; 3.12) ^d, f^	0.013
**CAL, mm**			
Baseline	4.95 (4.87; 5.12)	5.14 (4.85; 5.36)	0.331
1-month	4.31 (4.27; 4.47) ^b^	3.88 (3.75; 4.23) ^b^	0.058
3-months	3.94 (3.85; 4.13) ^c, e^	3.18 (3.06; 3.32) ^c^	0.049
6-months	3.59 (3.55; 3.71) ^d, g^	2.62 (2.58; 2.77) ^d, f, g^	0.015
**% sites with PD** ≥ **4 mm**			
Baseline	37.2 (34.9; 43.1)	36.7 (33.5; 39.5)	0.278
1-month	29.3 (25.4; 33.6) ^b^	28.1 (23.5; 31.2)	0.017
3-months	26.1 (22.4; 29.9) ^c^	21.1 (18.6; 24.3) ^c, e^	0.066
6-months	24.9 (23.8; 27.8) ^d^	18.2 (16.5; 23.6) ^d, f^	< 0.001
**BOP, %**			
Baseline	48.9 (43.6; 53.5)	47.8 (42.3; 56.3)	0.315
1-month	36.2 (33.6; 39.9) ^b^	29.6 (27.5; 35.6) ^b^	0.028
3-months	27.8 (24.6; 32.5) ^c, e^	20.6 (18.5; 23.8) ^c, e^	0.025
6-months	24.9 (21.3; 28.6) ^d, f^	18.2 (15.6; 22.9) ^d, f^	0.016
**PI, %**			
Baseline	37.5 (32.6; 41.2)	37.5 (33.1; 43.6)	0.331
1-month	32.9 (29.6; 34.6) ^b^	23.4 (20.6; 27.5) ^b^	0.027
3-months	25.2 (22.3; 28.6) ^c, e^	22.7 (20.6; 27.5) ^c, e^	0.105
6-months	23.2 (20.6; 26.3) ^d, g^	17.9 (13.6; 21.3) ^d, g^	0.036

^a^ no significance between baseline and 30 days; ^b^ significance between baseline and 30 days, ^c^ significance between baseline and 90 days; ^d^ significance between baseline and 180 days; ^e^ significance between 30 and 90 days; ^f^ significance between 30 and 180 days; ^g^ significance between 90 and 180 days. P-value significant < 0.017 (Bonferroni’s correction)

### Primary outcome

**Fig. 2:**
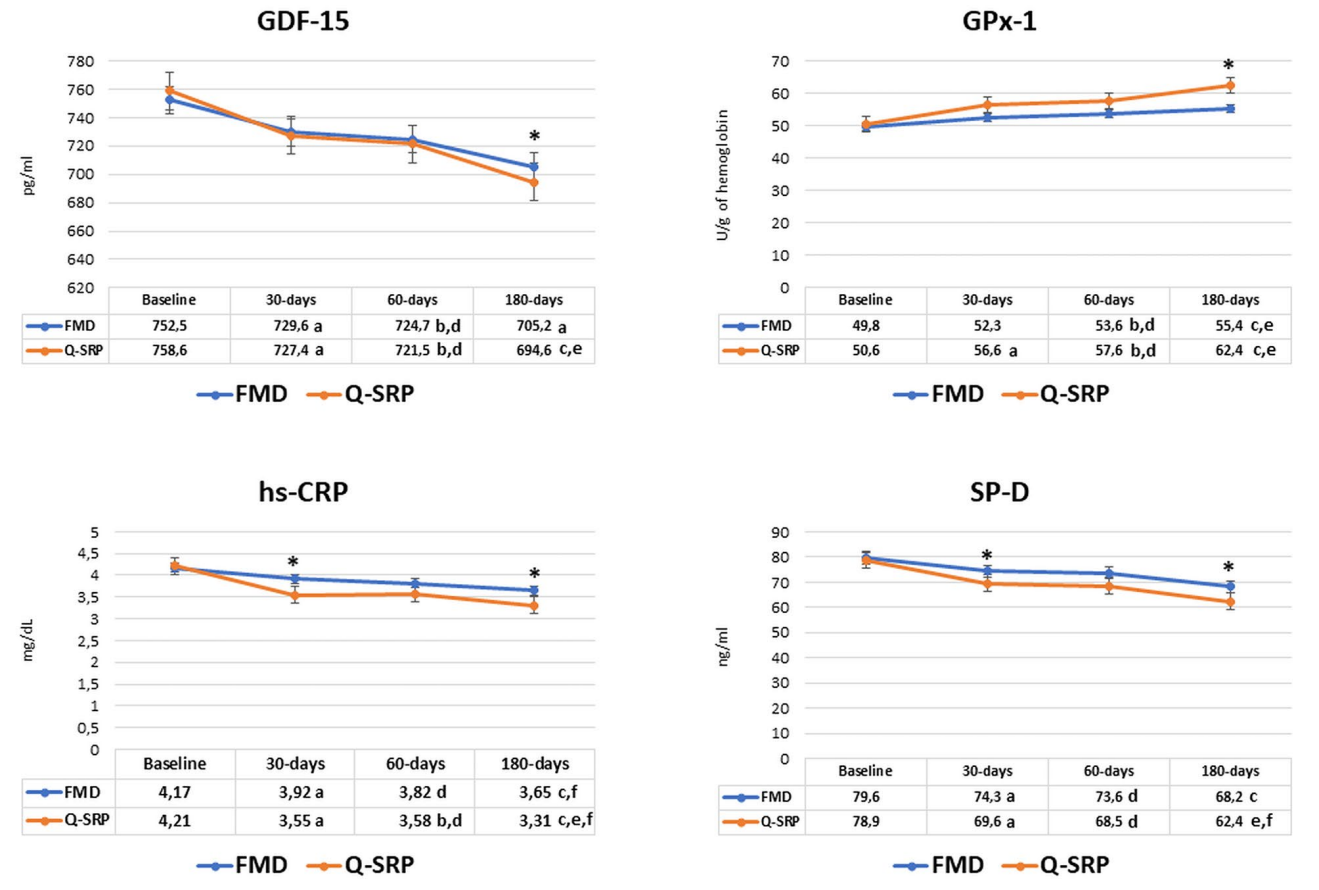
Differences among GDF-15, GPx-1, hs-CRP and SP-D at baseline and at each follow-up session. Results are expressed as mean and SD (standard deviation). a, significance between baseline and 30 days; b, significance between baseline and 90 days; c, significance between baseline and 180 days; d, significance between 30 and 90 days; e, significance between 30 and 180 days; f, significance between 90 and 180 days. * P-value significant < 0.008 (Bonferroni corrections). GDF-15, growth differentiation factor 15; GPx-1, glutathione peroxidase 1; hs-CRP, c-reactive proteins; SP-D, surfactant protein D

In comparison with the FMD group, at 6 months after therapy, the Q-SRP group showed a significant reduction of the GDF-15 levels (p = 0.012) (Table 2). Furthermore, in comparison with FMD, the Q-SRP group demonstrated a significant increase of GPx-1 levels (p = 0.011) and a reduction of hs-CRP (p = 0.019), and in SP-D (p = 0.015) concentrations levels at 6 months following therapy (Fig. 2).

At 6-months of therapy, the correlation analysis evidenced that there was a correlation between serum GDF-15 and smoking (p = 0.046), % sites with PD ≥ 4 mm (p = 0.022), number of teeth (p = 0.031), BOP (p = 0.044) and PI (p = 0.045). Serum GPx-1 levels were correlated with smoking (p = 0.044), HbA1c (p = 0.045), LDL-cholesterol (coeff. 0.196, p = 0.044), number of teeth (p = 0.041), CAL (p = 0.025) and BOP (p = 0.027); hs-CRP was correlated with HbA1c (p = 0.048), LDL cholesterol (p = 0.019), % sites with PD ≥ 4 mm (p = 0.032), BOP (p = 0.045), and PI (p = 0.039); SP-D levels were correlated with smoking (p = 0.025), number of teeth (p = 0.035); CAL (p < 0.001), BOP (p = 0.016), and PI (p = 0.011) (Table 3).

**Table 3:** Correlation analysis among GDF-15, GPx-1 and the analyzed variables at 6-months of treatment. For sex, males served as a reference. GDF-15, growth differentiation factor 15; GPx-1, glutathione peroxidase 1; hs-CRP, c-reactive proteins; SP-D, surfactant protein D. HbA1c, Glycated haemoglobin; BMI, Body Mass Index, PD, Probing Depth; CAL, Clinical Attachment Loss; BOP, Bleeding on Probing; PI, Plaque Index

Variable	GDF-15		GPx-1	
	*Rs coeff.*	*p-value*	*Rs coeff.*	*p-value*
**Age**	0.362	0.104	0.256	0.548
**Sex**	-0.155	0.335	0.331	0.317
**Smoking**	-0.108	0.046	-0.331	0.044
**Education**	-0.058	0.274	0.258	0.095
**HbA1c**	0.105	0.081	-0.258	0.045
**BMI**	0.205	0.663	0.309	0.524
**Total cholesterol**	0.105	0.048	0.201	0.332
**HDL cholesterol**	0.184	0.205	0.147	0.201
**LDL cholesterol**	-0.332	0.336	0.196	0.044
**% of sites with PD ≥ 4 mm**	0.305	0.022	-0.109	0.085
**Number of teeth**	-0.157	0.031	-0.215	0.041
**CAL (mm)**	0.274	0.065	-0.297	0.025
**BOP (%)**	0.356	0.044	-0.365	0.027
**PI**	0.269	0.045	-0.357	0.548
	**hs-CRP**		**SP-D**	
	*Rs coeff.*	*p-value*	*Rs coeff.*	*p-value*
**Age**	0.302	0.085	0.205	0.325
**Sex**	-0.128	0.105	-0.241	0.066
**Smoking**	-0.204	0.256	0.336	0.025
**Education**	-0.394	0.104	0.185	0.108
**HbA1c**	0.258	0.048	0.332	0.278
**BMI**	0.325	0.587	0.245	0.358
**Total cholesterol**	-0.258	0.056	-0.658	0.221
**HDL cholesterol**	0.365	0.065	0.365	0.365
**LDL cholesterol**	-0.274	0.019	0.478	0.258
**% of sites with PD ≥ 4 mm**	0.331	0.032	-0.108	0.069
**Number of teeth**	0.247	0.066	0.335	0.035
**CAL (mm)**	0.285	0.068	0.297	< 0.001
**BOP (%)**	0.369	0.045	-0.244	0.016
**PI**	-0.105	0.039	0.428	0.011

### Secondary outcome

The estimation of models aimed to determine the impact of Q-SRP and timing of treatment on GDF-15, GPx-1, hs-CRP, and SP-D concentration changes at 6 months using a two-way ANOVA test revealed that Q-SRP had a significant effect on the reduction of serum GDF-15 (p = 0.015), GPx-1 (p = 0.045), and SP-D (p = 0.045) together with the timing of treatment for GDF-15 (p = 0.028) and GPx-1 (p < 0.001) concentrations. More specifically, patients with high GDF-15, GPx-1, and SP-D baseline concentrations gained more benefits from Q-SRP at 6-month follow-up (Table 4).

**Table 4:** Results of two-way ANOVA for the dependent variable GDF-15, GPx-1, hs-CRP and SP-D. GDF-15, growth differentiation factor 15; GPx-1, glutathione peroxidase 1; hs-CRP, c-reactive proteins; SP-D, surfactant protein D

	GDF-15		
*Source of variation*	*MS*	*F*	*p-value*
**Group**	885.66	205.12	0.015
**Timing**	698.36	356.21	0.028
Group*Timing	21.24	3.35	0.002
**Within**	4.33		
	**GPx-1**		
*Source of variation*	*MS*	*F*	*p-value*
**Group**	265.55	136.12	0.045
**Timing**	247.36	105.41	< 0.001
Group*Timing	4.26	4.33	0.026
**Within**	3.28		
	**hs-CRP**		
*Source of variation*	*MS*	*F*	*p-value*
**Group**	389.36	196.54	0.105
**Timing**	205.56	158.45	0.331
Group*Timing	6.65	5.66	0.206
**Within**	2.41		
	**SP-D**		
*Source of variation*	*MS*	*F*	*p-value*
**Group**	415.41	205.44	0.026
**Timing**	248.66	306.56	0.226
Group*Timing	3.98	4.87	0.045
**Within**	2.66		

MS: Mean of Square. F: Fisher test; Group*Timing: Interaction term

## Discussion

In the last decade, an increasing amount of evidence has found that the chronic inflammatory stimulus linked to its pathogenic biofilm load is the real factor that directly connects periodontitis to many systemic diseases. Due to these factors, periodontal treatment, through its various approaches, has been shown to be of vital importance as a primary factor in managing the reduction of risk of development or aggravation of systemic diseases and endothelial damage. In this regard, a number of non-surgical instrumentation approaches that allow a stable reduction over time of possible early biomarkers of the risk of developing systemic chronic inflammatory diseases have been developed [22]. The purpose of the present study was to examine the effect of NSPT on systemic circulating biomarkers such as GDF-15 with GPx-1, hs-CRP and SP-D concentrations and to determine the impact of 2 NSPT approaches and timing on concentration changes of serum GDF-15, GPx-1, hs-CRP and SP-D at 6-months of therapy.

At 6-months of treatment, both groups of patients demonstrated, compared to baseline, a significant improvement of all analyzed biomarkers. However, compared to FMD, Q-SRP treatment produced a greater decrease in serum GDF-15, hs-CRP, and SP-D levels associated with increased GPx-1 levels. In this regard, several studies evidenced that, during periodontitis, imbalanced serum GDF-15 levels may influence the innate host defences against periodontal pathogens [17]. In this regard, it has been demonstrated that during the active phases of periodontitis, bacterial LPS have been proven to stimulate elevated serum GDF-15 levels via a process involving the synthesis of proteolysis products in gingival fibroblast [16]. Similarly, further investigations in a rat model of periodontitis showed that higher levels of serum GDF-15 were tightly correlated with the periodontal inflamed surface area already from the earliest stages of the disease [30].

However, the therapeutic significance in reducing several systemic CVD and inflammatory risk biomarkers among various non-surgical periodontal treatment approaches is still poorly understood.

In this regard, it has been shown a possible correlation between markers of low-grade periodontal inflammation and the etiology of CVD in patients with periodontitis, characterized by the periodontal microbial infection that stimulates the local and systemic production of pro-inflammatory cytokines such as TNF-, IL-1, and IL-6 [29, 30]. In fact, NSPT treatment through an intensive removal of subgingival dental plaque biofilms determined positive effects in blood lipid levels and decreased serum pro-inflammatory cytokine levels in individuals with periodontitis and hyperlipidemia [31]. Similarly, serum levels of IL-6 and IL-8 were dramatically decreased 6 months after periodontal treatment [32]. In this regard, some recent studies and meta-analyses [21, 33] confirmed the previous findings [34] that periodontal therapy influences clinical parameters and that there is no clinical variation across SRP methods.

Furthermore, the important clinical indicators of CVD include, among others, CRP, interleukin-6 (IL-6), lipid index, fibrinogen, tumor-necrosis factor-α (TNF-α) and GCF-15 [35]. More specifically, GCF-15 and CRP, a growth factor and an acute plasma protein, respectively, have been shown to act as a regulator in the early immune response during periodontitis [12, 36]. GDF-15, hs-CRP, and in SP-D have been shown to have a pro-inflammatory effect on endothelial cells [36] and are often used as a systemic inflammatory marker of CAD [37]. In this study, NSPT significantly decreased serum GDF-15, hs-CRP, and SP-D and increased GPx-1 in patients with periodontitis. GDF-15 and related markers have been reported to be a key inflammatory mediator released by activated macrophages in the intima [38] and a powerful inducer of CRP. In this regard, previous studies have reported that periodontal treatment can significantly decrease CRP levels and related inflammatory biomarkers in periodontitis patients [39, 40], while upregulating GPx-1 levels were related to reduced oxidative stress, inflammation, and bone loss in an experimental periodontitis model [41]. Furthermore, one prospective study showed that periodontal treatment determines a short-term positive reduction of several CVD biomarkers in patients with periodontitis and plays a crucial part in early endothelial risk dysfunction development [42]. However, other evidence has reported different conclusions on the influence of periodontal therapy on serum inflammatory risk biomarkers levels in periodontitis patients with uncertain effects of NSPT [34], especially in the long term [43].

In this regard, the present study has achieved significant results with Q-SRP approach with a positive correlation between serum GDF-15, GPx-1, hs-CRP and SP-D with the extent of periodontitis, evaluated through the association with the several periodontal parameters examined. Therefore, the reduction in serum GDF-15, hs-CRP and SP-D levels 6 months after periodontal therapy with Q-SRP implies that this therapy was able to determine a more effective breakdown of the bacterial biofilm and a superior host response compared to the FMD approach.

Furthermore, the two-way ANOVA analysis of the present investigation demonstrated that Q-SRP significantly improved and impacted serum GDF-15, GPx-1 and SP-D after six months of therapy. In addition, the same analysis demonstrated that at six months follow-up, patients who harboured high levels of GDF-15 and SP-D and low levels of GPx-1 before periodontal treatment positively benefited from the periodontal treatment efficacy at 180 days follow-up. In this regard, it has been demonstrated that periodontal treatment can induce a short-term inflammatory response resulting in a progressive and consistent reduction in systemic inflammatory biomarkers [44–48] and in an improvement of endothelial functions in patients with periodontitis [49]. In this regard, a large cohort population study in patients with periodontitis found an independent association between tooth loss and several prognostic biomarkers, suggesting that tooth loss and its underlying mechanisms may be involved in multiple pathophysiological inflammatory pathways implicated in the development and prognosis of CVD [11].

The present study had some limitations that should be addressed, such as the monocentric design and the short-term evaluation timing. More specifically, a longer follow-up and a higher number of enrolled patients would have been beneficial to assess the positive impact on differential clinical results for stable periodontal outcomes. Furthermore, a test group of CVD patients may have been needed to better determine the impact of periodontal treatment in reducing biomarkers linked with CVD. For these reasons, further analyses are required to better understand the benefits of non-surgical periodontal treatments on serum GDF-15 GPx-1, hs-CRP and SP-D.

In conclusion, the findings of the present randomized controlled trial demonstrated that, in all analyzed patients, both Q-SRP and FMD treatments were efficacious, at 6 months, in reducing periodontal parameters as well as serum CVD biomarkers. However, periodontal treatment performed with Q-SRP determined a better decrease in clinical periodontal parameters and improved GDF-15, GPx-1, hs-CRP and SP-D in patients with periodontitis. In addition, there is a tendency towards a more beneficial effect of periodontal treatment at 6 months if patients presented higher GDF-15 and SP-D and lower GPx-1 concentrations at baseline.

### Electronic supplementary material

Below is the link to the electronic supplementary material.

Supplementary Material 1: CONSORT Checklist of the study.

## Data Availability

Data are available from corresponding authors upon reasonable request.
